# Errata

**Published:** 1826-07

**Authors:** 


### THE LONDON MEDICAL AND PHYSICAL JOURNAL.

EDITED BY

RODERICK MACLEOD, M.D.

PHYSICIAN TO THE
WESTMINSTER GENERAL DISPENSARY,
AND LECTURER
ON THE THEORY AND PRACTICE OF PHYSIC, AND ON MATERIA MEDICA,
AT THE SCHOOL IN GREAT WINDMILL STREET.

(VOL. LVI.)
NEW SERIES, VOL. I.

Et quoniam variant morbi, variabimus artes;

Mille mali species, mille salutis erunt.

LONDON:

45589

JOHN SOUTER, 73, ST. PAUL'S CHURCH-YARD;

AND TO BE HAD OF ALL THE MEDICAL BOOKSELLERS.

1826.

W1
LO 31

*J. and C. Adlard, Printers,*
*Bartholomew Close.*

### INDEX

TO THE

FIRST VOLUME OF THE NEW SERIES

OF THE

### London Medical and Physical Journal.

PAGE

Absorbents, inflammation of, treated with Lunar Caustic . 228
Absorption, analysis of, Dr. Barry on . . . . 274
Acid, Prussic, observations on . 87
Address of the Editor on commencing a new Series . 1
Affections of the Brain . . 2, 10
Amenorrhœa, cases of, treated with Guaiacum . . 543
Amputation at the Knee-joint, case of . . . . 89
Anatomy of the Fœtal Brain, M. Tiedemann on the . . 364
——— Brain, Dr. Spurzheim on . . . 367
———, Morbid Plates of Dr. Hooper's . 560
Anatomical appearances in Gastritis . . . . . 82
Aneurism, Popliteal, remarkable case of . . . . 134
———, case of Subclavian, in which that artery was successfully tied . . . . 449
Animal Heat, Dr. Williams on . 360
——— Magnetism, Dr. Dupau on 463
Anus, artificial, operations for, 190, 285
Antimony, Tartarised, observations on . . . . 185
Antrum Maxillare, abscess of the 289
Artery, Carotid, wound of, with observations . . . . 331
Arteries, use of the probe in punctured wounds of . . 481
Artificial Pupil, observations on 38
Army, qualifications required of medical officers in the . 195
Arsenic, Dr. Christison on poisoning with . . . . 457
———, Dr. Venables on ditto 508
Associated Practitioners, their Report . . . . 197
Atrophia, its effects . . 566

PAGE

Back, Fungous Tumor of the . 208
Belladonna, on the external application of . . . 403
Beriberi, analysis of, Mr. Hamilton on . . . . 358
Bladder, on Sutures in wounds of the . . . . . 381
———, removal of Calculi from the . . . . . 572
Bilateral operation for Stone . 233
Bile, experiments on the use of the . . . . . 340
Biographical sketch of the late Mr. J. Pearson . . 51
Blood, analysis of Dr. Barry on 274
——— observations on . . 280
Books, Lists of, 95, 199, 295, 392, 487, 583
Brain, morbid appearances of, Dr. Mills on . . . . 74
——— Plates of . 560
Bronchocele, Mr. Earle's case of, in which the superior Thyroid Arteries were tied . . 201
Bronchi, case of Cherry-stone in the . . . . . 430

Calculi, removal of, from the Bladder . . . 572
Calculus removed from the female by dilating the Urethra, 214, 383, 384
———, large, removed from a child . . . . . 287
Carotid Artery, wound of . 331
Cataract, observations on . 178
Caustic, Lunar, application of, in Erysipelas, &c. . . 582
Censors of the Royal College of Physicians, observations on their conduct . . 390
Cherry-stone in the Bronchi, case of . . . 430
Chorea, case of . . . 342

PAGE

Coma following Salivation cured by reproducing Ptyalism . 231
Cupping, Mr. Kennedy on . 198

Dental Surgery, Mr. Koecker's 372
Diet, Dr. Paris on, analysis of, 157, 263
Digestion, Dr. Smith on . . 177
Diseases, Reports of prevalent, 92, 194, 291, 386, 483, 581
Distortions of the Spine, an account of the methods of treating, in Paris, *with engravings* . 489
Documents relative to Vaccination . . . . 143
Domestic Medicine, analysis of Dr. Graham's . . . 277
Duration of human life in France 88

Ectropium, new operation for . 572
Epiglottis, destruction of . 91
Ergot, observations on . . 87
Erysipelas, Dr. Stevenson on . 362
——— Mr. Travers' cases of 439
——— application of Lunar Caustic in . . . 582
Experiments on animals, by injecting putrid matters . 477
Exposition of the present state of the Medical Profession in Britain . . . . 369
Extirpation of the Ovarium 91, 578
——— Testicle . 482
Eye, remarks on representations of the . . . 38
Eyes of insects, their structure . 567

Feet, perspiration of the, M. Lobstein on . . . 180
Femur, cases of fractured . 312
Fever, Dr. Hewett on, 97, 241, 421, 538
——— cases of, with remarks, by Dr. Chambers . . 351
——— Dr. Black on, review of 450
——— Putrid, memoir on, by M. Bayle . . . 471
Follicular Ulceration of the Bowels in Fever . . 97, 241
Fœtus in Fœtu, case of . . 479
Fracture, case of ununited, of the Forearm, cured by excision of the ends of the bone . . 288
——— case of compound, in which a bitter infusion was used as a lotion . . . 438

Head, injuries of . . 210
Headache, Dr. Vaughan on . 563
Heat, animal, Dr. Williams on . 360

PAGE

Heart, cases of disease of the, 119, 219, 221
——— feigned diseases of . 290
——— wound of . . 578
Hernia, Strangulated, cases of, 15, 18, 23, 24, 26, 444, 446
Herpes Zoster, treated by the application of Caustic . . 233
Human life, duration of, in France 88
Hunterian Museum, observations on the admission to . 292, 390
Hydrocephalus, extraordinary case of . . . 86
Hydrocyanic Acid, observations on . . . . 87
Hydrophobia, analysis of Mr. White on . . . . 68
——— case of, by Mr. King . . . . 347
——— on the communication of . . . 374
Hyoscyamine, its effects . 566

Inflammation of the Absorbents, treated with Lunar Caustic . 228
——— Veins, observations on . . . 33
Injuries of the Head, cases of . 210
——— Thorax . 224, 528
Instrument for detecting animal matter in the atmosphere . 198
Institution, Medical Provident, of Scotland . . . 486
Intestines, Dr. Hewett on ulceration of the . . . 97
Iris, Dr. Watson on inflammation of the . . . . 359
Irritation, analysis of Mr. Travers on . . . . . 54
——— local, case of, by Mr. Earle . . . . 428

Jaw, amputation of, Dr. M'Clellan on . . . . . 188
———, lower, removal of the . 570
Journals, Meteorological, 96, 200, 296, 392, 488, 584

Kirronose, M. Lobstein on . 80
Knee-joint, amputation at the . 89

Lectures at the College of Physicians . . . . . 93
——— on the Medical Sciences delivered in London . . 388
Licentiates, letter on the jealousies of the . . . 484
Life, human, duration of, in France 88
Lime, Chlorate of, in Burns . 385
Linea Alba, rupture of . . 192

PAGE
List of the Lecturers in London 388
Lists of Books, 95, 199, 295, 392, 487, 583
Lithotomy, cases of, by MM. Dupuytren and Sanson . 233

Malformation of the Rectum . 204
Medical profession, present state of . . . . 369
——— and Physical Journal, plagiarism from . . . 483
——— Essays, Dr. Hall's, review of . . . . 547
Medicine, analysis of Dr. Graham's Domestic . . 277
Melanosis, analysis of Mr. Fawdington on . . . 271
Meteorological Journals, 96, 200, 296, 392, 488, 584
Monster, in whom the extremities were wanting . . 279
Morbid appearances of the Brain, analysis of Dr. Mills on . 74
Morphia, Acetate of, its use in Uterine Hemorrhage . . 479
Museum, Hunterian . . 390
Mustard Seed, analysis of Mr. Cooke on . . . 174

Nervous Circle which connects the Voluntary Muscles with the Brain . . . . 44
Neuralgia, case of, cured with Carbonate of Iron . . 545
Note respecting the case of James White . . . 195
Nux Vomica, observations on . 186

Observations on the Brain and Nerves in Monsters . . 85
——————— frequency of Follicular Ulceration of the Intestines in Fever . 97, 241
Œsophagus, perforations of the 569
Officers of the College of Physicians for the year 1826-7 . 485
Oleum Terebinthinæ in Purpura 582
Operation for the Piles, improved 574
Ophthalmia, Mr. Mackenzie on 317
———, analysis of Mr. Wardrop's paper on . . 356
Ovarium, extirpation of . 91, 578
———————, attempted by Dr. Granville . . 141

Pathology, M. Martinet's Manual of . . . . . 564
Phagedenic Ulceration, cases of, 122, 204
Phlebitis, observations on . 374
Phthisis, on the prevention of . 393
Phymosis, operation for . 90
Physicians, Lectures at the College of . . . 93
Physiology, analysis of Dr. Bostock's System of . . 276
Piles, improved operation for the 574
Placenta, new method of extracting the . . . 289
Popliteal Aneurism, Mr. Bell's case of . . . 134
Polypus of the Uterus, on the separation of . . . 432
Pregnancy, new species of extra-uterine . . . 80
Prevalent Diseases, Reports of the 92, 194, 291, 386, 483, 581
Prize question . . . 95
Probe, use of the, in wounds of Arteries . . . 481
Prussic Acid, observations on 87, 292
Pupil, artificial, remarks on . 38
Purpura, cases of . . 414
Ptyalism, cases of Coma following the sudden suppression of 231

Qualifications required in medical officers of the army . 195
Question, prize . . . 95

Rectum, malformation of . 204
Removal of the Lower Jaw . 570
Reports of prevalent Diseases, 92, 194, 291, 386, 483, 581
————— the Associated General Practitioners . . 197
Répertoire général d'Anatomie, &c. analysis of . 78, 553
Representations of the Eye, remarks on . . . 38
Respiration, Dr. Williams on . 360
Rheumatism, cases of, by Dr. Chambers . . 12, 114
———————, by Dr. Macleod . . . 14
—————, on Camphor in . 377
————— of the Heart, case of, cured by Acupuncture . 379
Recto-vesical operation for the Stone, cases of . . 233

Salivation, cases of Coma following . . . . 231
Scalp, M. Lisfranc's method of treating wounds of the . 526
Sloughing Ulceration, cases of, 122, 129, 132
Snails an article of food . 92
Specific properties of medicines, observations on . . 148

PAGE

Spina Bifida, case of, cured by puncture . . . 89
Stammering, note on . . 583
Stethoscope, Dr. Scudamore on, analysis of . . . 77
Strangulated Hernia, 15, 18, 23, 24, 26
Surgeons, College of, their new regulations . . . 386
Surgery, analysis of Mr. A. C. Hutchison on . . 166
Suture in wounds of the Bladder 381
Sympathy, analysis of Dr. Alison on . . . . . 252
Syphilis treated with scruple doses of Calomel . . . 436
———, treatment of, without Mercury . . . 482

Tartarised Antimony, observations on . . . 185
Testicle, cases of diseased, 209, 297, 446, 521
——— extirpation of . . 482
Thermometer, new division of the 294
Thigh-bone, remarks on the want of union in the neck of the . 504
Thorax, cases of injury of, with remarks . . . 528
Tracheotomy, case of, for the removal of a foreign body . 480
Transactions of the Medico-Chirurgical Society of Edinburgh, analysis of . . 356, 457
Transfusion, unsuccessful case of, 139
———, observations on the above . . . 293
Tumor, Fungous, on the Back 208
Turpentine, Dr. Wood on the use of, in a particular condition of Fevers . . . 182

PAGE

Ulceration, Follicular, Dr. Hewett on . . . 97, 241
———, Sloughing, Mr. Travers' cases of . . . 122
———, Mr. Green's ditto 129
———, Mr. Rose's ditto 132
———, Mr. Babington's ditto . . . . 204
Ununited Fracture, case of, cured by excision of the ends of the bone . . . . 288
Urinary Calculus, cases of, extracted from the female, by dilatation of the urethra . 214
Uterine Hemorrhage, use of Acetate of Morphia in . . 479
———, case of, unsuccessfully treated by Transfusion . . . 139
Uterus wanting . . . 278
———, Polypus of, separation of 432

Vaccination, note on . . 582
———, documents relative to . . . . . 143
———, Dr. Maraschini's experiments on . . . 285
———, Dr. Gregory on . 410
Variolous Epidemic in Sweden . 283
Viscera, case of transposition of the . . . . 345

White Mustard Seed, analysis of Mr. Cooke on . . 174
Wound of the Heart . . 578

Yellow Fever, observations on 187

CRITICAL ANALYSES . *Pages* 54, 157, 252, 356, 453, 547
COLLECTANEA . . . 85, 177, 278, 374, 471, 566
INTELLIGENCE . . . 92, 194, 285, 387, 483, 581
METEOROLOGICAL REGISTER 96, 200, 296, 392, 488, 584

### NAMES OF AUTHORS,

*Of whose Works, Observations, &c. either a detailed Account, or more or less general View, is given in the present Volume.*

| | PAGE |
|---|---|
| AGER, Dr. his letter to the Editor | 391 |
| ALLAN, Mr. on Specifics | 148 |
| ALISON, Dr. analysis of his paper on Sympathy | 252 |
| ANDRAL, M. (fils) on Chronic Gastritis | 82 |
| BABINGTON, Mr. on Sloughing Ulceration | 204 |
| BARRY, Dr. analysis of his Researches on the Blood, Absorption, &c. | 274 |
| BAYLE, M. on Putrid Fever | 471 |
| BELL, Mr. C. on the Nervous Circle | 44 |
| ——— case of Popliteal Aneurism | 134 |
| ——— cases illustrative of the treatment adopted at the Middlesex Hospital in Fractures of the Femur | 312 |
| ——— cases of Injury of the Thorax, with remarks | 224, 528 |
| BOSTOCK, Dr. analysis of his System of Physiology | 276 |
| BOYLE, Mr. case of Hernia | 23 |
| ——— cases of Syphilis treated with scruple doses of Calomel | 436 |
| BLACK, Dr. on Fever, review of | 453 |
| BRESCHET, M. on a new species of Extra-uterine Pregnancy | 80 |
| BRODIE, Mr. cases of Hernia | 24, 25 |
| ——— case of Fungous Tumor on the Back | 208 |
| ——— case of Disease of the Testicle | 209 |
| ——— Paper, with Cases, on the Testicle | 297, 521 |
| CHAMBERS, Dr. case of Cerebral Affection | 5 |
| ——— cases of Rheumatism | 12, 114 |
| ——— Fever, with remarks | 351 |
| ——— Purpura | 419 |
| CHEVALIER, Mr. case of Urinary Calculus removed from the Female by dilating the Urethra | 214 |
| ——— on the external application of the Extract of Belladonna | 304 |
| CHRISTISON, Dr. on Poisoning with Arsenic, &c. | 457 |
| DENDY, Mr. case of Rupture of the Linea Alba | 192 |
| DUPAU, Dr. review of, on Animal Magnetism | 463 |
| DUPUYTREN, Mr. cases of Bilateral Operation for Stone | 241 |
| ——— case of successful Operation for Subclavian Aneurism | 449 |
| EARLE, Mr. case of Bronchocele, in which the superior Thyroid Arteries were tied | 201 |
| ——— case of Malformation of the Rectum | 204 |
| ——— diseased Bone removed from the Thigh | 428 |
| FAWDINGTON, Mr. analysis of his case of Melanosis | 271 |
| GONDRET, Dr. on Cataract | 178 |
| GRANVILLE, Dr. his instrument for detecting animal matter in the atmosphere | 198 |
| ——— case in which extirpation of Ovarian Tumors was attempted | 141 |
| ——— documents relative to Vaccination | 143 |
| GREEN, Mr. cases of Sloughing Ulceration | 129 |
| ——— case of Strangulated Ventral Hernia | 444 |
| GREGORY, Dr. on Vaccination | 410 |
| GUTHRIE, Mr. observations on Inflammations of Veins | 33 |
| HALL, Dr. MARSHALL, review of his Medical Essays | 547 |
| HAMILTON, Mr. analysis of his paper on Beriberi | 358 |
| HAWKINS, Dr. case of Cerebral Affection | 7 |
| ——— Nervous Affection | 221 |
| ——— cases of Purpura Hemorrhagica | 414 |
| HEWETT, Dr. his remarks on Fever | 97, 241, 421, 538 |
| HIGGINBOTTOM, Mr. cases of Inflammation of the Absorbents, treated by the application of Caustic | 228 |
| HOOPER, Dr. his Plates of the Brain | 560 |
| HUTCHISON, Mr. A. C. on Surgery, analysis of | 166 |
| ——— case of Imperforate Anus | 285 |
| HUNTER, Mr. case of Strangulated Exomphalos | 446 |

PAGE
JEFFREYS, Mr. cases of Fractured Spine . . . 28, 30, 31
——— Injury of the Head . . . . 210
——— Disease of the Testicle . . . . 446
JEWEL, Mr. case of unsuccessful Transfusion . . . . 139
——— cases of Amenorrhœa successfully treated with Guaiacum 543
KING, Mr. on Spurred Rye . . . . . . . . 87
KOECKER, Mr. his Dental Surgery . . . . . 372
LATHAM, Dr. P. M. case of Cerebral Affection . . . . 2
LOBSTEIN, M. on Kirronose . . . . . . . . 80
——— on Sweating of the Feet . . . . . 180
MAC ANDREW, Dr. case of Chorea . . . . . . 342
MACKENZIE, Mr. Professor, remarks on the Eye . . . 38
———, on Ophthalmia . . . . . 317
MACLEOD, Dr. cases of Rheumatism . . . . . 14
——— Coma following the sudden suppression of Ptyalism 231
MACMICHAEL, Dr. cases of Diseases of the Heart - . . . 119
MARTINET, Dr. his Pathology . . . . . . . . 564
MAYO, Mr. H. experiments on the use of the Bile . . 340
——— remarks on the Want of Bony Union in the Neck of the Thigh-bone . . . . . . . . . . 504
M'CLELLAN, Dr. on Amputation of the Jaw . . . . 188
MILLS, Dr. on the Brain, analysis of . . . . . . 74
NICHOLL, Dr. W. on Phthisis . . . . . . . 393
——— his note on the Ol. Terebinthinæ in Purpura Hemorr. 582
PARIS, Dr. on Diet, analysis of . . . . 157, 263
PEARSON, Mr. J. Biographical Sketch of . . . . . 51
QUAIN, Mr. his translation of Martinet's Pathology . . 564
RECAMIER, Dr. (of Paris,) case of Cerebral Affection . . . 10
ROSE, Mr. cases of Sloughing Ulceration . . . . . 132
——— case of Transposition of the Viscera . . . 345
——— Compound Fracture, in which a bitter infusion was used as a lotion . . . . . . . . . 438
SANSON, M. cases of Lithotomy . . . . . . 230
SCUDAMORE, Dr. on the Stethoscope, analysis of . . . 77
SHAW, Mr. case of Hernia . . . . . . 18, 26
——— Injury of the Thorax . . . . . 227
——— note on Mr. King's case of Hydrophobia . . . 350
——— account of the Methods of treating Curvatures of the Spine adopted in Paris . . . . . . . . . 489
SMITH, Dr. NATHAN, on Amputation at the Knee-joint . . 89
——— on Extirpation of the Ovarium . . . 91
——— on Digestion . . . . . . . 177
SPURZHEIM, Dr. his Anatomy of the Brain . . . . . 367
——— Illustrations of Phrenology . . . . 369
STEVENSON, Dr. on the Contagious Nature of Erysipelas . . 362
STONE, Mr. on the separation of the Neck of Polypus of the Uterus . 432
TIEDEMANN, M. on the Brain and Nerves in Monsters . . 85
TRAVERS, Mr. on Constitutional Irritation, critical analysis of . 54
——— case of Strangulated Hernia - . . . . . 15
——— cases of Sloughing Ulceration . . . . 122
——— case of Wound of the external Carotid Artery . . 331
——— cases of Traumatic Erysipelas . . . . 439
TWEEDALE, Mr. his note respecting the case of James White . 195
VAUGHAN, Dr. notice of his Essay on Headaches . . . 563
VENABLES, Dr. on the Detection of Arsenic and Corrosive Sublimate 508
WARDROP, Mr. analysis of his paper on Ophthalmia . . 356
WATSON, Mr. on Inflammation of the Iris . . . . . 359
WEBSTER, Dr. his case of Cherry-stone in the Bronchi . . 430
WHITE, Mr. on Hydrophobia, analysis of . . . . . 68
WILLIAMS, Dr. on Respiration and Animal Heat . . . . 360
WOOD, Dr. on Oil of Turpentine in a particular condition of Fever . 182
YOUNG, Dr. case of Disease of the Heart . . . . . 219

THE LONDON
### Medical and Physical Journal.

N° 329, VOL. LVI.] JULY, 1826. [N° 1, *New Series.*

THE LONDON MEDICAL AND PHYSICAL JOURNAL *professedly contains an account of the most important improvements which are made in Medicine and the collateral branches of Science: and it has been thought, that, in addition to the means hitherto adopted for conveying this information, considerable advantage would be deduced from giving each month a history of such* CASES *occurring at* PUBLIC INSTITUTIONS, *as might seem calculated to illustrate any points in pathology or practice. On mentioning this idea to the Physicians and Surgeons connected with some of the principal Hospitals and Dispensaries in the metropolis, the utmost readiness was manifested by these gentlemen to have an account of the cases under their care laid before the public; while every facility was promised for obtaining the necessary information. The Editor begs respectfully to express his sense of the liberality which has thus been shown by his professional brethren; and, while he absolves them from all responsibility with regard to the accuracy of the details, he pledges himself to take every means in his power of ascertaining the authenticity of the cases which he publishes: at the same time he requests it to be understood, that he will be ready to correct any misstatement, should such inadvertently be made.*

*The manner in which it appears to the Editor most eligible to arrange these, is according to subjects,—giving in succession a few cases of the same disease, particularly where there is any difference in the symptoms or treatment, the detail of which is likely to prove instructive. It is obvious that this object cannot be accomplished in every instance; but, with a view to secure its fulfilment as frequently as possible, it is intended to make the Reports retrospectively, not confining them exclusively to those cases which are of recent occurrence.*

*The reader will perceive that some other changes have been made in the contents of the Journal, which it is hoped will be regarded as improvements.*

*There is one circumstance which will be perceived with regret: it is the retirement of* MR. BACOT. *The Editor, however, has pleasure in being able to state, that, although that gentleman's avocations will prevent him from taking the same responsible share as heretofore in the management of the Journal, the reader will not be entirely deprived of his valuable services.*

R. MACLEOD.

*Henrietta-street, Cavendish-square;*

*June* 10, 1826.

### MONTHLY LIST OF MEDICAL BOOKS.

Practical Observations in Surgery; more particularly as regards the Naval and Military Service. By Alex. Copland Hutchison. Second Edition, enlarged.

An Exposition of the State of the Medical Profession in the British Dominions.

Experimental Researches on the Influence exercised by Atmospheric Pressure upon the Progression of the Blood in the Veins. By David Barry, m.d.

Recherches Expérimentales sur les Causes du Mouvement du Sang dans les Veines. Par David Barry, m.d.

Farther Remarks on Hernia, in Explanation of the Nature of Strangulation, and of Obliterated Intestine, &c. By E. Geoghegan, m.r.c.s.

Transactions of the Medico-Chirurgical Society of Edinburgh. Vol. II. With Plates.

An Essay on Cupping, &c. &c. By Charles Kennedy, Surgeon.

Observations on the Efficacy of White Mustard Seed, in Affections of the Liver, Internal Organs, and Nervous System. By Charles Turner Cooke, Surgeon.

The Surgeon-Dentist's Anatomical and Physiological Manual. By G. Waite, Member of the Royal College of Surgeons.

Pinnock's Catechism of Anatomy.

### METEOROLOGICAL JOURNAL,

### NOTICE TO CORRESPONDENTS.

*The present Number affords a specimen of the Arrangement which it is our intention to adopt hereafter. We may remark, that, on the present occasion, the* Cases *have run somewhat longer than we anticipated, and, as a necessary consequence, the* Collectanea *has been abridged to a corresponding extent.*

*The Profession in general, and our old Correspondents in particular, are respectfully invited to favour us with Cases, Facts, and information of any kind connected with Medicine and the collateral Sciences.*

*We have to acknowledge the receipt of Communications from* Mr. Higginbottom, Dr. Granville, Mr. Harrold, Dr. Smith, Mr. Allen, Mr. Jewel, Dr. Alexander, and Mr. Tod.

*The case of* Unsuccessful Transfusion *in our next.*

