# Errata

**Published:** 1826-08

**Authors:** 


					errata..
Unavoidable circumstances led to the preceding Nnmber being passed rapidly through the
press, which irave rise to several typographical errors.?Page 1, for 44retrospectively," read
' retrospective."

				

